# Research Progress on Sex Pheromone Receptors in Insects

**DOI:** 10.3390/insects17040382

**Published:** 2026-04-01

**Authors:** Henan Ju, Youmiao Li, Baolin Ou, Wanqiu Huang, Huifeng Li, Yongmei Huang, Yanqing Li, Tianyuan Chen, Jinfeng Hua

**Affiliations:** 1 Department of Sweet Potato Genetic Breeding and Application, Institute of Maize Research, Guangxi Academy of Agricultural Sciences, Nanning 530007, China; juhenan123@163.com (H.J.); 19978672869@163.com (Y.L.); oubaolin1203@163.com (B.O.); wanqiu8311@163.com (W.H.); 605lifeng@163.com (H.L.); huangyongmei322@163.com (Y.H.); liyq2004.7@163.com (Y.L.); tianyuanchen@126.com (T.C.); 2 College of Agriculture, Guangxi University, Nanning 530004, China

**Keywords:** insects, sex pheromones, sex pheromone receptors, genetic identification, functional verification

## Abstract

Insect sex pheromone receptors (PRs) are crucial for regulating mating and reproduction. In the insect olfactory perception pathway, the pheromone-binding protein (PBP) facilitates the efficient translocation of sex pheromones, enabling them to bind to PRs. PRs convert chemical signals into electrical signals, which are transmitted to the insect central nervous system to ultimately regulate reproductive behaviors. Thus, conducting functional analysis of PRs not only clarifies the molecular mechanism underlying insect mating via sex pheromone recognition and reveals the intrinsic regulatory link between sex pheromone detection and mating behavior but also provides theoretical support for the scientific understanding of the insect olfactory system. Additionally, this research lays a core theoretical foundation for the development of green pest control technologies in agriculture and forestry. This paper systematically reviews the research methods, technical principles, and advantages and disadvantages of techniques used to study insect *PR* genes. It summarizes representative identified PRs and their corresponding research strategies, aiming to provide a reference for future investigations into insect chemical communication and for the advancement of pest control practices.

## Simple Summary

Sex pheromone receptors (PRs) are critical chemical sensors in insects that govern mating and reproduction. This review synthesizes current knowledge on PRs, focusing on their recognition mechanisms, methods for their identification and functional validation, and their structural features. These insights not only deepen our understanding of insect olfactory communication but also provide a conceptual and methodological foundation for developing environmentally friendly pest control strategies. Interfering with PR function offers a promising avenue to disrupt pest mating, thereby reducing reliance on chemical pesticides and promoting sustainable agricultural and forestry practices.

## 1. Introduction

Chemical communication is a fundamental biological strategy for insects to mediate intraspecific and interspecific interactions, underpinning key behaviors such as mating, foraging, and predator avoidance [1]. Insects are capable of synthesizing and detecting a diverse array of semiochemicals—including aggregation, alarm, trail, and sex pheromones—each of which serves distinct ecological functions [2]. Among these, sex pheromones stand out as the primary chemical signals driving species-specific mating in most insect taxa [3,4]. Their highly specific recognition not only determines individual mating success and population persistence but also acts as a critical molecular barrier for reproductive isolation among closely related species, shaping the formation and maintenance of insect species diversity [5]. Antennae is the primary olfactory organ and in many insect species, females release sex pheromones to elicit attraction by the males [6], the accurate detection of sex pheromones is the prerequisite for this specialized communication, and this process hinges on a key functional class of molecules in the insect olfactory system: sex pheromone receptors (PRs) [7]. The maxillary palps, as one of the olfactory organs of insects, may also be involved in the recognition of PRs [8]. Dissecting the molecular characteristics, identification methods, and regulatory mechanisms of PRs is thus essential to unraveling the molecular basis of insect sex pheromone communication, while also providing a core molecular target for the development of green pest control technologies that disrupt insect reproductive behavior [9,10].

The insect olfactory system is the primary sensory pathway for detecting volatile chemical signals such as sex pheromones and host volatiles, evolving remarkable specificity and sensitivity through long-term adaptive evolution [11]. It is composed of a suite of functionally complementary gene families, including odorant receptors (ORs), ionotropic receptors (IRs), gustatory receptors (GRs), odorant-binding proteins (OBPs), and chemosensory proteins (CSPs) [12,13,14]. Among these, the OR family is the central player in recognizing volatile odorants, orchestrating a range of vital insect behaviors—from mating and foraging to aggregation and anti-predator responses—via selective binding to distinct odorant ligands [15]. PRs represent a highly specialized functional class of the OR family, differing from general ORs that recognize a broad spectrum of environmental volatiles by their adaptation to sex pheromone detection [7]. PRs do not form a single, conserved monophyletic clade across all insect orders; their evolutionary relationships are clade-specific, with distinct PR lineages evolving independently in different insect groups [16]. Structurally, most PRs possess a typical seven α-helical transmembrane domain architecture, a feature shared with classical G protein-coupled receptors (GPCRs), but with a reversed membrane topology—an intracellular N-terminus and an extracellular C-terminus—that distinguishes them from mammalian GPCRs [17]. A small number of PRs, such as *Holotrichia parallela* (Coleoptera: Scarabaeidae) HparOR14, have been predicted to contain six transmembrane domains via in silico modeling, though this structural characterization awaits biochemical validation [18]. For all insect ORs, including PRs, functional signal transduction is dependent on the assembly of heteromultimeric complexes with the highly conserved odorant receptor co-receptor (Orco) [19,20,21,22,23,24,25]; this interaction is a prerequisite for PRs to anchor to the plasma membrane of olfactory sensory neurons (OSNs) and initiate downstream signaling.

**Figure 1:**
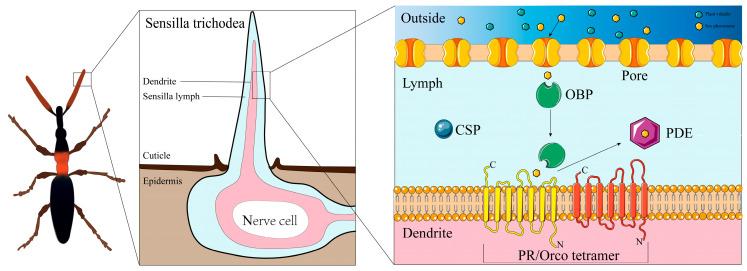
Schematic diagram of the PR-mediated olfactory signal transduction pathway in insects.

The molecular process of sex pheromone detection by PRs involves precise coordination of multiple components, beginning with the capture of sex pheromone molecules in the insect antennae. Pheromone-binding proteins (PBPs), abundant in the antennal sensilla, bind to lipophilic sex pheromone molecules in the aqueous sensillar lymph and facilitate their delivery to PRs on the OSN plasma membrane, a role that supports the efficient initiation of neuronal electrical signals (Figure 1) [26,27]. Upon reaching the PRs, sex pheromone molecules interact with the receptor’s ligand-binding pocket, triggering a conformational change that initiates signal transduction [7]. While PRs are primarily specialized for detecting conspecific sex pheromones, their recognition is not strictly exclusive: many PRs can respond to multiple closely related pheromone components, and a small subset may also be activated by specific plant volatiles [16,28]. This interaction converts extracellular chemical signals into intracellular electrical signals, which are transmitted to the insect central nervous system to regulate mating behavior [29]. PRs are responsible for the detection of sex pheromone signals at the peripheral sensory level; true perception of these signals occurs in the insect brain [30].

Research into insect sex pheromones and their receptors has advanced dramatically since the first isolation and identification of the *Bombyx mori* (Lepidoptera: Bombycidae) sex pheromone by Butenandt in 1959 [31]. In the years that followed, sex pheromones from a range of insect taxa, such as *Diprion similis* (Hymenoptera: Diprionidae) and *Anomala rufocuprea* (Coleoptera: Scarabaeidae), were sequentially analyzed and validated [32,33], and the identification and functional characterization of the first insect PR, *B*. *mori* BmorOR1, in 2004 marked a pivotal milestone, opening the door to systematic studies of PR molecular biology [34]. Since then, high-throughput omics technologies and bioinformatics tools have accelerated PR identification across diverse insect species [35], revealing a wealth of PR sequences and functional characteristics [36]. However, this research landscape is marked by a pronounced taxonomic bias: Lepidoptera PRs have been extensively characterized, with cumulative genomic, transcriptomic, and functional data forming a relatively comprehensive understanding of their evolution and function [36]. In stark contrast, PR research in other major insect orders remains fragmented and underdeveloped [18]. Current research also faces unresolved challenges: the evolutionary drivers of PR structural and functional diversity across insect orders remain unclear, the molecular basis of PR ligand-binding plasticity is poorly understood, and there is a lack of validation for how laboratory-based functional findings translate to the complex chemical environments insects encounter in nature, where factors like temperature, humidity, and background odors may modulate PR function [37,38].

As a core molecular node mediating insect sex pheromone communication, PR research carries important theoretical significance and broad application potential [9,10]. Interfering with PR-mediated sex pheromone recognition can effectively disrupt pest mating and population growth, thereby reducing agricultural economic losses [39]. Yet the field still grapples with key unresolved questions: the PR–ligand recognition mechanism is not fully elucidated [40], functional verification systems lack standardization across taxa, and structural biology insights have not been fully integrated with in vivo functional evidence [7]. To address these gaps, this review systematically synthesizes the research progress of insect PR identification, structural analysis, and functional verification technologies, critically comparing the advantages and limitations of different methodological approaches. To enhance the comprehensiveness of this review, we also focus on the relevance across different insect taxa, emphasizing that the research scope is not limited to Lepidoptera—despite the current research focus on PRs being heavily weighted toward lepidopteran species. We highlight the evolutionary conservation and lineage-specific divergence of PR sequences and structural features across insect orders, explicitly address the taxonomic bias and technical bottlenecks in current research, and propose future development directions rooted in cutting-edge technological advances. By consolidating existing knowledge and identifying critical research gaps, this review aims to provide a structured and insightful reference for further exploring the molecular mechanisms of insect olfactory communication and for guiding the innovation of PR-targeted green pest control strategies for sustainable agricultural and forestry management.

## 2. Identification of Insect PRs

PRs are core regulators of insect reproductive behavior, and the efficient, accurate identification of *PR* genes forms the fundamental basis for all subsequent structural and functional research [7]. With the rapid advancement of high-throughput omics technologies and bioinformatics analysis tools [41], the screening and identification pipeline for *PR* genes has been continuously refined. This process can be divided into three sequential, mutually corroborating stages, each playing a unique role in the precise discovery of *PR* genes.

### 2.1. Preliminary Screening of Candidate PR Genes Based on Genomic/Transcriptomic Sequencing

Advances in genomics and transcriptomics have significantly improved the efficiency of screening and identifying PRs across diverse insect taxa (Table 1) [42]. As primary technical methodologies for constructing candidate *PR* gene libraries, genomic sequencing and transcriptomic sequencing follow a standardized workflow [43,44]: first, high-quality whole-genome data or antennal transcriptome datasets are generated; next, in silico screening is performed to identify potential PR candidate sequences from the *OR* gene family. Subsequently, gene cloning techniques are applied to obtain the full-length coding sequence (CDS) of the target gene, and qRT-PCR assays are conducted to characterize its spatial expression patterns in antennae (with a focus on male antennae), which enables the preliminary confirmation of its candidacy as a PR. However, this antennae-expression-based identification method has limitations. It cannot rule out false positives, as some ORs highly expressed in antennae may not be functionally involved in pheromone recognition, and it may also miss PRs with low expression levels or tissue-specific expression outside antennae [45]. For example, in research focusing on *Spodoptera frugiperda* (Lepidoptera: Noctuidae), investigators leveraged whole-genome sequencing (WGS) and RNA sequencing (RNA-seq) data to screen for *OR* genes that are highly expressed in male antennae. Following this, via gene cloning and verification of expression profiles, they successfully identified several PRs, such as the well-characterized *SfruOR6* [46,47]. Similarly, in *Galleria mellonella* (Lepidoptera: Pyralidae) research, the *PR* gene *GmelOR4* was identified using a genomic database and the same technical approach [48].

### 2.2. Bioinformatics Analysis for Targeted Narrowing of PR Candidates

Bioinformatics tools are essential for screening candidate genes with conserved structural features from massive omics datasets, significantly boosting identification efficiency [88]. Common bioinformatics tools, their functions, and application scenarios are shown in Table 2. Sequence alignment tools (e.g., BLAST) can infer the functional association between new genes and known PRs through homology analysis [89]. Protein structure prediction tools (e.g., AlphaFold) provide a basis for three-dimensional structure modeling [90]. Gene expression analysis tools (e.g., DESeq2) help evaluate tissue-specific expression patterns, supporting the screening of candidates enriched in antennae [91]. In *S. frugiperda* studies, researchers used the BLASTn program to search the genome for PR homologous sequences and screened candidates with specific gene family domains via HMMER software (v3.0), ultimately identifying *SfruOR6* [92]. In research focusing on *Micromelalopha troglodyta* (Lepidoptera: Notodontidae), investigators employed the ORFfinder to analyze transcriptomic datasets, identification of 74 *OR* genes de novo, eight of which were classified as PR candidates after integrating criteria like homology to known PRs and enriched expression in male antennae, consistent with the PR screening logic applied in *S. frugiperda* studies [93].

Homology-based prediction is an efficient initial screening method, yet it has notable limitations [44,94]. Sequence homology does not perfectly match receptor function, and minor amino acid variations at key sites can lead to significant differences in ligand recognition specificity [95]. Existing reference databases show an obvious bias toward Lepidoptera, with a lack of receptor data for Coleoptera, Hemiptera and other orders, which markedly reduces predictive reliability for non-lepidopteran species [40]. Receptor genes have complex evolutionary characteristics such as rapid expansion/contraction and functional differentiation [96], making it difficult for traditional homology analysis to accurately reflect true functional evolutionary relationships. Therefore, this method can only provide a preliminary screen and cannot replace subsequent experimental verification.

### 2.3. Phylogenetic and Expression Profile Analysis for Confirming PR Candidate Credibility

Phylogenetic analysis and tissue-specific expression profiling serve as pivotal experimental evidence for validating the reliability of candidate PR genes [97,98]. These two approaches integrate evolutionary and expression-based evidence, forming a dual-evidence framework for screening candidate *PR* genes.

#### 2.3.1. Phylogenetic Analysis

Phylogenetic analysis plays a pivotal role in the preliminary functional prediction of candidate PRs. By constructing phylogenetic trees, typically based on PR amino acid or nucleotide sequences, researchers can elucidate the interspecific evolutionary relationships of PRs across different insect taxa [99]. For instance, in *S. frugiperda*, phylogenetic analysis based on amino acid sequence alignments showed that the identified PRs exhibit high sequence homology with well-characterized PRs from other lepidopteran species, suggesting they perform conserved sex pheromone recognition functions [100]. As demonstrated in research on *Dendrolimus punctatus* (Lepidoptera: Lasiocampidae), based on amino acid sequence alignment and functional validation of XOE-TEVC, it was demonstrated that this species has significant differentiation from the identified lepidopteran PR lineage, forming an independent evolutionary branch, and the PR protein in this lineage specifically recognizes the Type I pheromones [68].

#### 2.3.2. Expression Profile Analysis

Tissue-specific expression profiling acts as key experimental evidence for the spatial localization of candidate *PR* genes [101], with its core principle lying in the fact that most PRs are highly expressed in insect male antennae a canonical tissue expression trait that distinguishes PRs from non-*PR* genes. Molecular biology techniques, including qRT-PCR, in situ hybridization, and RNA in situ hybridization, are used to detect the expression levels and spatial distribution of candidate *PR* genes across various tissues (antennae, heads, thoraxes, abdomens, legs, and wings) [63,102,103]. High enrichment in male antennae is adopted as the core screening criterion to validate the reliability of these candidate PRs [104]. The central value of this step is that it leverages the tissue-specific expression features of PRs to exclude non-*PR OR* genes with either non-antenna expression or low antennal expression, thereby pinpointing candidate genes whose spatial expression specificity aligns with the functional characteristics of PRs. For instance, in the identification of most lepidopteran *PR* genes, such as *B. mori BmorOR1* and *H. armigera HarmOR6*, qRT-PCR assays have confirmed high expression of these candidate genes in male antennae, providing pivotal evidence for verifying their identity as PRs [34,57].

### 2.4. Current Status of PR Gene Identification and Sequence Evolutionary Characteristics in Insects

PRs have been increasingly identified across a broad range of insect taxa. Representative examples include *Drosophila melanogaster* (Diptera: Drosophilidae), *Helicoverpa armigera* (Lepidoptera: Noctuidae), and *Plutella xyllostella* (Lepidoptera: Plutellidae)*,* for which PRs have been functionally validated following identification, reflecting the significance of PR-mediated recognition of sex pheromones (Table 1). Among insect taxa, lepidopteran PR research is the most mature, with cumulative genomic, transcriptomic, and functional data supporting a relatively comprehensive understanding of this gene family’s diversity and evolution [40]. By contrast, PR identification in other major orders is still significantly lagging. In 2012, the *PR* gene (*McOr3*) of the first Coleoptera insect was identified. The number of functionally validated PRs in Coleoptera remained relatively small [86]. This highlights a long-standing gap in our understanding of PRs in non-lepidopteran insects, indicating considerable untapped research potential.

*PR* gene sequences exhibit both conservation and species-specificity across diverse insect species [105]. In terms of conservation, PRs harbor signature conserved domains, and the majority of PR family members possess a 7TM-GPCR-like topology. These conserved structural features serve as the molecular basis for their signal transduction function in mediating sex pheromone recognition [106,107]. Additionally, specific conserved motifs are present in the amino acid sequences of PRs across different insect taxa [40]. This sequence-level conservation directly reflects the evolutionary stability of their core functional domains, as these conserved regions are typically associated with key functions such as ligand binding and signal transduction—ensuring the conservation of PR-mediated sex pheromone recognition during evolution [108]. From the perspective of specificity, notable diversity exists in the amino acid sequences of PRs across taxa. This divergence is functionally driven, endowing PRs with the capacity to discriminate and bind to sex pheromones with structurally distinct functional groups (Table 1). Such specificity ensures the accuracy of species-specific mating communication, which is a key evolutionary adaptation for maintaining reproductive isolation [16,28]. For example, BmOR1 from *B. mori* specifically recognizes (E,Z)-10,12-hexadecadienol (bombykol) [34]; while a single-base mutation in the *PR* genes of *Ostrinia furnacalis* (Lepidoptera: Crambidae) and *Ostrinia nubilalis* (Lepidoptera: Crambidae) confers specificity in sex pheromone recognition, leading to natural behavioral isolation despite laboratory hybridization capacity [16].

This synergistic “Conservative-Diversity” evolutionary pattern of PRs has key biological significance: conserved domains maintain core signal transduction function to ensure basic sex pheromone recognition activity, while specific sequence variations enable adaptation to distinct sex pheromone structures, forming species-specific “sex pheromone–PR” recognition systems [109]. This prevents the erroneous cross-recognition of non-conspecific sex pheromones, safeguarding reproductive isolation. Furthermore, by leveraging comparative genomics to characterize the expansion and contraction patterns of *PR* gene families across diverse species, researchers can decipher the regulatory principles underlying adaptive evolutionary changes in these gene families—providing insights into how PRs evolve to match species-specific ecological and reproductive needs [11,110,111]. This knowledge is critical for in-depth analysis of insect olfactory signaling and for formulating PR-based sustainable pest control strategies.

### 2.5. The Collaborative Relationship Among the Complete Identification Systems and the Contributions of Each Stage

The three stages of insect *PR* gene identification—omics-based preliminary screening, bioinformatics-driven targeted refinement, and phylogenetic and expression profiling validation—are not isolated. Instead, they form a systematic identification framework characterized by sequential progression, mutual corroboration, and multi-layered evidence accumulation. The synergistic integration of high-throughput sequencing, systematic bioinformatics analyses, and in-depth evolutionary analysis has significantly driven the field of insect PR identification [35]. In the future, this systematic workflow will continue to enable efficient and accurate PR identification. Furthermore, integrating structural biology techniques such as cryo-electron microscopy will allow for a deeper analysis of PR structure–function relationships.

Based on the above identification and functional studies, further analysis of PR protein structures is critical to reveal their working mechanisms.

## 3. Structural Analysis of PR Proteins

### 3.1. Analysis of Secondary Structure

As a specific functional category of ORs, PRs exhibit the classic structural features of ORs. Conserved domain analysis is one of the common methods for identifying PRs [112]. The research on specific recognition and binding of ligands suggests that extracellular loops (ECLs) play a crucial role. A well-characterized case is BmOR1: The second extracellular loop (ECL-2) of this protein contains four conserved proline residues (Pro-165, Pro-178, Pro-181, Pro-194). These residues maintain the stability of the binding pocket. In *Heliothis virescens* (Lepidoptera: Noctuidae) HvirOR13, *Diaphania indica* (Lepidoptera: Crambidae) DindOR1, and *Plutella xylostella* (Lepidoptera: Plutellidae) PxylOR1, there are 2 proline residues in their ECL-2 that are highly conserved with Pro-165 and Pro-194 of BmorOR1. Site-directed mutagenesis confirmed that substitutions of Pro-165 or Pro-194 significantly reduce the binding affinity of BmOR1 to its sex pheromone, demonstrating the critical roles of these conserved residues in ligand–receptor recognition [113]. Studies have shown that the amino acid composition and arrangement pattern in other insect PRs also possess similar characteristics to this [114].

The transmembrane domains are involved in the ligand binding process and can also directly regulate the transduction efficiency [115]. Proteomic-based analyses of the transmembrane domain provide critical functional insights. For example, the *D. melanogaster* pheromone receptor DmelOR67d, which mediates recognition of male-produced pheromones: its individual transmembrane α-helices contain 20–30 hydrophobic residues, and the seven-transmembrane assembly forms a barrel-like cavity that serves as a core ligand-binding region. Site-directed mutagenesis studies reveal that substituting the evolutionarily conserved Pro-238 residue with leucine diminishes DmelOR67d’s ligand sensitivity by 2.7-fold. It is worth noting that this residue is also highly conserved in PRs of *B. mori*, highlighting that conserved residues in transmembrane domains modulate ligand–receptor interactions and signal transduction efficiency [116]. These findings confirm that conserved amino acid residues in the transmembrane region are core components maintaining PR structural stability and functional activity.

Beyond ECLs and transmembrane domains, the ligand-binding pocket and ion channel structure of PRs exert indispensable impacts on their functional performance [117]. Mechanistically, these structural elements directly participate in core processes such as ligand capture, binding specificity determination, and ion conductance during signal transduction—collectively modulating the efficiency and specificity of PR-mediated sex pheromone recognition [118]. The research team headed by Guirong Wang elucidated key structural determinants of PR function by comparing the molecular mechanisms of HarmOR11 and HarmOR13, two well-characterized PRs from the *H. armigera*. Their findings demonstrated that the three-dimensional shape and spatial size of the PRs’ ligand-binding pocket (LBP) are the primary factors dictating the structural compatibility of sex pheromones with the receptor, thus defining which pheromone types can be physically accommodated. Additionally, the amino acid composition within the LBP (e.g., residues involved in hydrogen bonding, hydrophobic interactions) directly regulates the specificity of ligand–receptor recognition and the stability of the resulting PR–pheromone complex, which are critical for efficient signal transduction [119]. The gating of ion channels constitutes a pivotal step in signal transduction cascades mediated by PRs. Conformational changes in the ion channel structure not only dictate the efficacy of channel opening (e.g., open probability and duration) but also directly modulate ion flux magnitude—two parameters that synergistically regulate the initiation, amplification, and propagation of neural signals in the olfactory pathway [120].

Collectively, insect PRs possess conserved amino acid residues and secondary structural elements that form the basis for their sex pheromone recognition and signal transduction functions. Structural differences in the LBP determine PR recognition specificity, making the prediction and analysis of PR secondary structures a key link in deciphering their functional mechanisms.

### 3.2. Analysis of Three-Dimensional Structure

For the decipherment of 3D structures, Cryo-Electron Microscopy (Cryo-EM) and X-ray Crystallography stand as the two most widely utilized structural biology approaches to date [121]. Cryo-EM has witnessed unprecedented breakthroughs in the structural characterization of biomacromolecules in recent years [122,123]. Unlike X-ray crystallography, which requires protein crystallization, Cryo-EM allows for high-resolution structural determination of proteins under near-physiological states, preserving their native conformation. This makes Cryo-EM an optimal platform for dissecting the 3D architecture of membrane-bound receptors like PRs. For instance, Zhao et al. [124] employed single-particle Cryo-EM to determine the 3D structure of the AaOR10/Orco complex, revealing a tetrameric assembly mode and clarifying the molecular activation mechanism. Butterwick et al. [125] characterized the 3D structure of an Orco homologue from *Apocrypta bakeri* (Hymenoptera: Pteromalidae) using Cryo-EM, elucidating its inherent tetrameric architecture and identifying that odorant binding induces conformational changes leading to pore opening.

X-ray crystallography is used to determine the 3D structure of proteins by analyzing the X-ray diffraction patterns generated by protein crystals [126]. It has shown great potential but, to date, no X-ray crystal structures of insect PRs have been published. However, its application in related systems, such as determining the structure of the *Euplotes raikovi* (Ciliophora: Euplotidae) pheromone Er-13 [127], suggests that it may play a role in future PR research.

The studies above underscore that Cryo-EM is a powerful tool for deciphering the 3D structures of insect olfactory proteins. These techniques enable the elucidation of key conformational dynamics and molecular mechanisms, providing structural insights indispensable for understanding PR-sex pheromone recognition and for designing targeted pest control strategies.

From the perspective of research trends, predicting protein structures based on amino acid sequences and characterizing PRs functions have become the focus of future studies [128]. Propelled by technological advancements, researchers can further explore the conservation and divergence of PR structural features across different insect lineages, as well as their adaptive significance in the course of evolution. It is noteworthy that the number of resolved G protein-coupled receptor (GPCR) structures from 2020 to 2022 has surpassed the cumulative total from 2000 to 2019, underscoring the rapid progress of structural biology techniques [129]. This progress is primarily attributable to breakthroughs in applying Cryo-EM to resolving GPCR complexes. This trend strongly indicates that Cryo-EM will become the primary means for investigating the three-dimensional structure of PRs in the future [122,123,130]. Analyzing the amino acid composition and three-dimensional conformation of PRs is expected to profoundly reveal their functional mechanisms, laying a theoretical foundation for developing new insect behavior regulation strategies.

## 4. Functional Verification of PR Proteins

Elucidating the structural characteristics of PRs provides a basis for understanding their working modes, whereas systematic functional verification is indispensable to confirm their actual biological roles. Functional verification of PRs relies on multiple complementary strategies [131]. XOE-TEVC and cell line-based heterologous expression are widely used for in vitro ligand recognition and activation analysis of PRs. *Drosophila* transgenic expression systems, including the empty neuron system (Δab3A), enable in vivo functional analysis of PRs in native olfactory neurons. RNA interference achieves transient gene silencing to verify in vivo functions, while CRISPR/Cas provides definitive genetic evidence via stable gene editing. These methods form a systematic platform from in vitro characterization to in vivo validation, jointly revealing the physiological roles of PR proteins in sex pheromone communication.

### 4.1. Xenopus Oocyte Expression and Two-Electrode Voltage Clamp Recording System (XOE-TEVC)

The *Xenopus* oocyte expression and two-electrode voltage clamp recording system (XOE-TEVC) is a classic technique for in vitro functional validation of PRs [50,51,52,53,54,55,56,57,58,59,60,61]. It enables heterologous expression of *PR* genes in oocytes to record electrophysiological responses to sex pheromones. XOE-TEVC offers distinct advantages: *Xenopus* oocytes are large (diameter 1–2 mm), facilitating microelectrode insertion and stable recording [132]; the expression system exhibits high stability and enables efficient expression of exogenous PR proteins, ensuring sufficient receptor levels for reliable testing [133,134].

A potential limitation is the endogenous expression of muscarinic receptors in oocytes, which can lead to non-specific activation; however, this can be controlled with appropriate pharmacological blockers and control experiments [135]. Despite this, XOE-TEVC has been widely applied in functional studies on PRs across various insect species, such as *Orthaga achatina* (Lepidoptera: Pyralidae) and *Apolygus lucorum* (Hemiptera: Miridae) (Table 1).

### 4.2. Drosophila Transgenic Expression Approach

Among the most widely used heterologous platforms for characterizing insect ORs and PRs, the *Drosophila* ab3 “empty neuron” system [136,137] leverages the fly’s genetically accessible olfactory system. The system uses engineered OSNs as in vivo “cellular chassis” to express exogenous receptors, eliminating endogenous receptor interference while preserving intact signal transduction, enabling reliable electrophysiological detection of receptor-ligand interactions [138]. In addition, the T1 trichoid sensillum system is a specialized platform designed for the functional study of PRs, which has been well-established and validated in Drosophila research: this system is centered on the T1 trichoid sensilla housing the ORNs expressing the sex pheromone receptor Or67d, and through genetic replacement of endogenous Or67d with exogenous PRs, it enables direct assessment of heterologous PR function in a physiological context dedicated to pheromone perception, with functional readout also achieved via single sensillum recording (SSR) [81].

The ab3 system is based on the ab3A OSNs located in the basiconic sensilla of *Drosophila* antennae [139]. In wild-type flies, ab3A neurons express endogenous receptors OR22a and OR22b. In the engineered system, these endogenous receptors are knocked out to generate “empty” neurons with intact signal transduction machinery but no olfactory responsiveness. Exogenous ORs/PRs can be specifically expressed in ab3A neurons via the OR22a-GAL4/UAS system, and their functions are characterized using SSR [136,140]. This system is highly versatile, suitable for functional analysis of various ORs and PRs, capable of providing a physiologically relevant cellular environment, and featuring mature, highly sensitive and stable detection methods [141]; however, due to potential compatibility differences between exogenous receptors and *Drosophila*’s endogenous signaling pathways, the response efficiency of some receptors may be impaired, and the system cannot fully simulate the complex multi-receptor cooperative ligand recognition in native insects. In comparison, the T1 sensillum system, with its specialization for pheromone signal transduction, offers a more physiologically relevant background for PR characterization, though its application is relatively focused on pheromone receptor research rather than the broad spectrum of olfactory receptors applicable to the ab3 system.

### 4.3. Cell Line Heterologous Expression System

Heterologous expression systems, such as the Human Embryonic Kidney 293 (HEK293) cell line and *S. frugiperda* (SF9) cell line, serve as important platforms for the in vitro functional verification of PRs [48,80]. These systems enable heterologous expression of PRs in mammalian or insect cells. When combined with calcium imaging techniques, cellular responses to pheromones can be measured, verifying functional characteristics like ligand specificity and response intensity [142]. For example, when investigating *H. armigera* PRs (HvOR13, HvOR14, HvOR16), researchers used HEK293 cells for heterologous expression and found that these receptors specifically responded to the sex pheromone component (Z)-11-hexadecenal (Z11-16:Ald) [57]. Zboray et al. [143] co-expressed *OR* genes and Orco in multiple cell lines. They then used the GCaMP6 fluorescent calcium indicator to detect changes in intracellular calcium concentration, successfully verifying the functions of seven insect ORs and identifying their corresponding specific ligands.

The cell line-based heterologous expression system offers multiple advantages: they can achieve efficient expression of PRs by optimizing expression conditions; they enable real-time, dynamic monitoring of PR activation; and both insect and mammalian cell lines provide a post-translational modification environment close to the native state, facilitating correct protein folding. For instance, EposOR3 from *Eriocrania semipurpurella* (Lepidoptera: Eriocraniidae) heterologously expressed in HEK293 cells exhibited high sensitivity to its sex pheromone [144]. Similarly, MdesOR115 from the *Mayetiola destructor* (Diptera: Cecidomyiidae) can exhibit specific responsiveness to the sex pheromone component (2S,8E,10E)-8,10-tridecadien-2-yl acetate when heterologously expressed in the same HEK293 cell system [80].

In cell line selection, three key factors—intrinsic traits of the PR, experimental purpose, and cost—should be considered. Insect cell lines (e.g., Sf9) more closely mimic the native physiological environment, making them ideal for analyzing natural functions. Mammalian cell lines (e.g., HEK293), by contrast, offer the advantages of simple operation and short culture cycles, thus being more suitable for high-throughput functional screening of PRs (Table 3). Collectively, cell-based heterologous expression systems provide a robust platform for exploring PR functional properties.

### 4.4. RNA Interference

RNA interference (RNAi) is a reverse genetics approach that mediates the specific silencing of target genes via the introduction of double-stranded RNA(dsRNA) [145]. By inducing the degradation of mRNA encoding target PRs, this technology suppresses the protein expression of the receptors; changes in insect behavior are then monitored to verify the functional significance of the target PRs. In *Ectropis obliqua* (Lepidoptera: Geometridae), RNAi silencing of the *PR* gene *EoblOR17* significantly reduced the insect’s mating rate, directly confirming its key role in mating behavior [146]. In *Grapholita molesta* (Lepidoptera: Tortricidae), oral administration of *GmolOR2*-targeting dsRNA effectively downregulates the gene’s expression and significantly reduces male adults’ sensitivity to sex pheromones [74]. Similarly, in the *A. lucorum*, RNAi-mediated silencing of the *PR* gene AlucOR4 significantly impaired the insect’s responsiveness to its key sex pheromone components [147].

RNAi provides several key advantages: high specificity, allowing for precise targeting of individual *PR* genes [148,149]; flexible dsRNA delivery methods (microinjection, oral feeding, topical application) [150]; and strong environmental compatibility, as dsRNA is easily degraded under natural conditions, aligning with green pest control concepts [151,152].

Nevertheless, this technology faces practical challenges. On the one hand, dsRNA is highly susceptible to degradation by endogenous and environmental nucleases, leading to unstable silencing efficacy and short duration of effect, often requiring repeated delivery [37,153]. On the other hand, there are significant interspecific differences in dsRNA absorption efficiency; some insects have intestinal barriers (e.g., thick peritrophic matrix, tight junctions) that hinder dsRNA from reaching target tissues, limiting silencing effects (Table 3) [154]. Furthermore, dsRNA is prone to rapid degradation under field conditions due to UV radiation, rainfall, and microbial activity [155]. To tackle these issues, researchers have developed nanoparticle-based delivery systems, such as chitosan nanoparticles, which encapsulate dsRNA to enhance its stability and cell penetration, thereby significantly boosting RNAi efficiency [156]. With the ongoing optimization of dsRNA delivery systems, RNAi technology is expected to assume a more prominent role in both the functional analysis of PRs and the regulation of pest behaviors.

### 4.5. CRISPR/Cas Gene Editing Technology

CRISPR/Cas gene editing technology, originating from bacterial and archaeal adaptive immune systems, uses guide RNA (gRNA) to direct Cas nucleases to cleave DNA at specific sites [157]. This enables precise gene knockout, knock-in, or site-directed mutagenesis, making it an irreplaceable genetic manipulation tool in biomedicine, agriculture, and related fields [158,159,160]. This system has evolved beyond Cas9-based to include variants Cas12a and Cas13a, achieving more efficient and precise genomic modification capabilities [161].

For in vivo PR functional studies, CRISPR/Cas technology produces gene knockout or mutant insects by targeted editing of insect *PR* genes. Subsequent observation of their behavioral and physiological changes enables clarification of PR functional mechanisms. For example, in *Spodoptera littoralis* (Lepidoptera: Noctuidae), CRISPR-Cas9 knockout of the *PR* gene *SlitOR5* significantly weakened male moths’ electrophysiological responses to the major sex pheromone component Z9,E11-14: OAc, reduced courtship and mating behaviors, and specifically shrank the volume of the MGC glomerulus responsible for processing this pheromone signal. This confirms SlitOR5’s key role in both pheromone perception and associated neural development [118]. For *Cydia pomonella* (Lepidoptera: Tortricidae), targeted knockout of the *PR* gene *CpomOR1* via CRISPR-Cas9 significantly decreased pest fecundity, with eggs laid by female moths failing to hatch normally [162].

CRISPR/Cas technology offers three key advantages: high editing efficiency enables rapid construction of PR function-deficient strains; strong precision—optimized sgRNA design effectively reduces off-target risk [163]; and stable heritability of editing effects facilitates long-term functional studies [164]. However, it still faces limitations: off-target effects cannot be fully eliminated, which may undermine the accuracy of phenotypic analysis [165,166]; and gene editing systems are still immature for some insects, with technical operations like embryo microinjection proving particularly difficult (Table 3) [132]. As the CRISPR/Cas system undergoes ongoing optimization and new editing tools emerge. It will not only strengthen research on insect chemical communication mechanisms but also provide robust support for developing green pest control strategies.

## 5. Conclusions and Future Perspectives

### 5.1. Conclusions

As a functionally specialized subset of the insect OR family, PRs play a key role in regulating insects’ mating and reproductive behaviors by specifically detecting sex pheromones [27]. This paper systematically reviews the identification systems, structural analysis progress, and functional verification methods of PRs. It highlights current research limitations—uneven taxonomic coverage, ambiguous PR multifunctionality, high verification costs, and RNAi field application bottlenecks—and offers a theoretical framework to support future research and green pest control efforts.

### 5.2. Future Perspectives

Aligning with the practical needs of green pest control and advances in research on insect chemical communication, future studies on insect PRs should prioritize the following key directions:

(1) **Expanding systematic excavation of *PR* gene resources:** Future work should address the current bias toward lepidopteran species by integrating advanced sequencing technologies (e.g., third-generation transcriptome [167], nanopore sequencing [168] and HiFi sequencing [169]) to capture full-length PR transcripts in key coleopteran and hemipteran pests. Furthermore, single-cell transcriptome sequencing of antennae holds significant promise [170], enabling precise correlation of specific OSNs with the PRs they express and the pheromone components they detect. Integrating these approaches with conserved domain screening and antenna-specific expression analysis will help construct PR gene maps for non-lepidopteran pests and establish a comprehensive cross-order PR database.
(2) **Deepen research on multifunctional recognition mechanism of PRs:** Current studies mostly focus on PRs’ role in sex pheromone recognition, yet pests also need to respond to host volatiles, suggesting PRs may have “dual-function” characteristics. Future work should XOE-TEVC and GC-MS technology to systematically evaluate PR response spectra to both pheromones and host volatiles. RNAi can be used to silence target PRs and observe changes in mating and oviposition preferences, clarifying their role in multi-signal integration and filling research gaps on synergistic regulation of multi-source chemical signals.
(3) **Optimize PR function verification technology system:** Current technologies like XOE-TEVC and cell line have high operational costs [18], making them insufficient to meet large-scale screening needs. In the future, priority should be given to developing miniaturized electrophysiological recording equipment to reduce detection costs and to constructing a PR ligand prediction model based on amino acid sequences and structural characteristics to reduce the workload of experimental verification and provide technical support for rapid functional annotation.
(4) **Advance analysis of PR structure and evolutionary mechanisms**: With Cryo-EM resolution reaching 1.8 Å [130], resolving the three-dimensional structure of the PR–Orco complex bound to sex pheromones will clarify the impact of key amino acid variations in the binding pocket on recognition specificity. Combined with comparative genomics, this will help systematically analyze sequence and structural differences across insect orders, revealing the evolutionary principles of “sex pheromone-PR” matching systems and providing new perspectives on the molecular mechanisms of interspecific isolation.
(5) **Construct a field control system based on PR interference**: As a core PR-targeted approach, RNAi faces challenges including rapid dsRNA degradation in pests [37,38] and low delivery efficiency. Future work should develop new delivery systems (e.g., chitosan–rosin composite nanoparticles [152]) to enhance dsRNA stability and cell penetration. Future work should also establish a dsRNA expression system mediated by transgenic crops, integrate CRISPR/Cas technology to develop PR knockout strains, systematically evaluate the impact of PR interference on pest population dynamics and ultimately develop a synergistic control technology system combining “PR interference and sex pheromone trapping [171],” reduce reliance on chemical pesticides, and support the sustainable management of agricultural and forestry ecosystems.

## Figures and Tables

**Table 1:** Insect part PRs, ligands, and functional verification methods.

Insect Order	Species	PRs	Sex Pheromone	Function Verification Method	References
Lepidoptera	*Bombyx mori*	BmorOR1, BmorOR3, BmorOR4-6	(E,Z)-10,12-hexadecadienol	XOE-TEVC	[34,49]
	*Scopula subpunctaria*	SsubOR9, SsubOR53, SsubOR55	(Z,Z)-3,9-cis-6,7-epoxy-nonadecadiene	XOE-TEVC	[50]
	*Heliothis virescens*	HvirOR6, HvirOR13, HvirOR15, HvirOR16	Z9-16: Ald	XOE-TEVC	[51,52,53]
	*Ostrinia nubilalis*	OnubOR3, OnubOR6	Z11- and E11-14: OAc	XOE-TEVC	[54]
	*Ostrinia furnacalis*	OfurOR4, OfurOR6-8, OfurOR5b	Z/E12-14: OAc	XOE-TEVC	[55]
	*Mythimna separata*	MsOR1	Z9-14: Ac	XOE-TEVC	[56]
	*Helicoverpa armigera*	HarmOR6, HarmOR11, HarmOR13-14b, HarmOR15-16	(Z)-9-hexadecenal (Z9-16: Ald) and (Z)-11-hexadecenal (Z11-16: Ald)	XOE-TEVC	[57,58,59,60]
	*Agrotis segetum*	AgseOR3-5	(Z)-5-decenyl, (Z)-7-dodecenyl	XOE-TEVC	[61]
	*Spodoptera litura*	SlituOR1, SlituOR3	Z9E11-14: OAc	RNAi	[62,63]
	*Spodoptera* *frugiperda*	SfruOR6, SfruOR11, SfruOR13, SfruOR16, SfruOR56, SfruOR 62	Z9,E12-14: OAc	XOE-TEVC	[47]
	*Plutella xylostella*	PxylOR1, PxylOR6, PxylOR41, PxylOR45, PxylOR59	Z11-16: Ald, Z11-16: Ac	XOE-TEVC, *Drosophila* OR67d neurons	[45,56,64,65]
	*Phthorimaea operculella*	PopeOR1, PopeOR3	(E,Z)-4,7-13: OAc and (E,Z,Z)-4,7,10-13: OAc	XOE-TEVC	[66]
	*Spodoptera exigua*	SexiOR13	Z9, E12-14: OAc	XOE-TEVC	[67]
	*Dendrolimus punctatus*	DpunOR45-46	(Z)-5-dodecenol	XOE-TEVC	[68]
	*Epiphyas postvittana*	EposOR1, EposOR6-7, EposOR30, EposOR34, EposOR45	(E)-11-tetradecenyl acetate	XOE-TEVC	[69]
	*Loxostege sticticalis*	LstiPR2	E11-14: OAc	XOE-TEVC	[70]
	*Cydia pomonella*	CpomOR3	8,10-Dodecadien-1-ol	XOE-TEVC	[71]
	*Galleria mellonella*	GmelOR4	nonanal and 2-phenylacetaldehyde	HEK293	[48]
	*Mamestra brassicae*	MbraOR16	Z11-16: Ac	-	[72]
	*Cnaphalocrocis medinalis*	CmedPR1, CmedOR27	Z11-18: OH and Z11-18: Ald	XOE-TEVC	[73]
	*Grapholita molesta*	GmolOR2	Z8-12: OAc	XOE-TEVC	[74]
	*Operophtera brumata*	ObruOR1	1,3Z,6Z,9Z-19: H	XOE-TEVC	[75]
	*Orthaga achatina*	OachPR1-5	Z11-16: OH	XOE-TEVC	[76]
Hymenoptera	*Campoletis chlorideae Uchida*	CchlOR18, CchlOR47	14: Ald and 2-Hep	RNAi	[77]
	*Cephus cinctus*	CcinOR2i	9-acetyloxynonanal	-	[78]
	*Harpegnathos saltator*	HsOR36	octacosane	-	[79]
Diptera	*Mayetiola destructor*	MdesOR115	(2S,8E,10E)-8,10-tridecadien-2-yl acetate	HEK293	[80]
	*Drosophila melanogaster*	DmelOR67d	11-cis Vaccenyl Acetate (cVA)	-	[81]
	*Bactrocera dorsalis*	BdorOR88a	ME	XOE-TEVC	[82]
Hemiptera	*Apolygus lucorum*	AlucOR40, AlucOR77	(E)2-hexenyl butyrate	XOE-TEVC	[83,84]
	*Adelphocoris lineolatus*	AlinOR33	trans-2-hexenyl butyrate	XOE-TEVC	[85]
Coleoptera	*Holotrichia parallela*	HparOR14	Methyl L-isoleucinate and (R)-(−)-linalool	RNAi	[18]
	*Megacyllene caryae*	McOR3, McOR5	(S)-2-methyl-1-butanol and 2-phenylethanol	XOE-TEVC	[86]
	*Ophraella communa LeSage*	OcomOR2, OcomOR4, OcomOR12	5-tricosene	-	[87]

**Table 2:** Commonly used bioinformatics analysis tools.

Tools	Function
BLAST (https://blast.ncbi.nlm.nih.gov/; accessed on 25 March 2026)	Used for sequence alignment to determine the homology between newly discovered receptor genes and other known genes, thereby inferring their functions
ClustalW v2.1/MUSCLE v5.3.3	Used for multiple sequence alignment to analyze the differences and conserved regions of receptor gene sequences among different species or different individuals of the same species and reveal evolutionary relationships
MEGA v12.1.2/PhyML v3.3.20250515	Used for conducting multi-sequence alignment and phylogenetic analysis of insect gene/protein sequences, constructing highly accurate phylogenetic trees and completing evolutionary feature analysis, as well as topological structure reliability verification Through this, it reveals the evolutionary relationships, conservation patterns and differentiation rules of genes among different groups
Phyre2 v2.2/SWISS-MODEL (https://swissmodel.expasy.org/, accessed on 26 March 2026)/AlphaFold v3	Used for protein structure prediction, predicting the three-dimensional structure of receptors through homology modeling, providing a basis for molecular docking and functional analysis
AutoDock v4.2.6/Vina v1.2.6/PyMOL v3.1.6.1	Used for molecular docking, simulating the interaction between receptors and sex pheromone ligands, evaluating binding affinity and binding mode, thereby understanding the ligand recognition mechanism of receptors
DESeq2 v1.50.2/edgeR v4.8.2	Used for gene expression analysis, studying the expression levels of receptor genes in different tissues, different developmental stages or different physiological conditions, and understanding their biological functions and regulatory mechanisms
MEME v5.5.9/HOMER v4.11	Used for conserved element identification, analyzing the cis-acting elements in the promoter region of receptor genes, predicting the binding sites of transcription factors, and revealing the molecular mechanism of gene expression regulation
EBImage v4.52/GraphPad v11/Origin v10.3	Used for image processing to assist phenotypic analysis
TMHMM v2.0	Used for predicting the transmembrane domains of proteins
ORFfinder (https://www.ncbi.nlm.nih.gov/orffinder/, accessed on 25 March 2026)	Used for predicting the Open Reading Frames of genes

Notes: BLAST (Basic Local Alignment Search Tool) and ORFfinder were obtained from the National Center for Biotechnology Information (Bethesda, MD, USA, https://blast.ncbi.nlm.nih.gov/, accessed on 25 March 2026; https://www.ncbi.nlm.nih.gov/orffinder/, accessed on 25 March 2026); ClustalW v2.1 and MUSCLE v5.3.3 were provided by the European Molecular Biology Laboratory (EMBL, Hinxton, UK, http://www.clustal.org/, accessed on 26 March 2026) and https://www.drive5.com/muscle/, accessed on 26 March 2026; MEGA v12.1.2 (Molecular Evolutionary Genetics Analysis) was from https://www.megasoftware.net/, accessed on 26 March 2026 and PhyML v3.3.20250515 was from http://www.atgc-montpellier.fr/phyml/, accessed on 26 March 2026; Phyre2 v2.2 was from Imperial College London (London, UK, http://www.sbg.bio.ic.ac.uk/phyre2, accessed on 26 March 2026), SWISS-MODEL was from the University of Basel (Basel, Switzerland, https://swissmodel.expasy.org/, accessed on 26 March 2026), and AlphaFold v3 was from DeepMind Technologies Ltd. (London, UK, https://deepmind.google/en/science/alphafold/, accessed on 26 March 2026); AutoDock v4.2.6 and Vina v1.2.6 were developed by The Scripps Research Institute (La Jolla, CA, USA, https://autodock.scripps.edu/, accessed on 26 March 2026), and PyMOL v3.1.6.1 was developed by Schrödinger, LLC (San Diego, CA, USA, https://pymol.org, accessed on 26 March 2026); DESeq2 v1.50.2 and edgeR v4.8.2 were cited from Harvard T.H. Chan School of Public Health (Boston, MA, USA, https://bioconductor.org/packages/release/bioc/html/DESeq2.html, accessed on 26 March 2026) and Walter and Eliza Hall Institute of Medical Research (Parkville, Australia, https://bioconductor.org/packages/release/bioc/html/edgeR.html, accessed on 26 March 2026); MEME v5.5.9 were cited from University of Queensland (Brisbane, Australia, https://meme-suite.org, accessed on 26 March 2026) and HOMER v4.11 form University of California (Oakland, CA, USA, http://homer.ucsd.edu, accessed on 26 March 2026); EBImage v4.52 was from Bioconductor (Oxford, UK, https://bioconductor.org, accessed on 26 March 2026), GraphPad Prism v11.0 was from GraphPad Software (San Diego, CA, USA), and Origin v10.3 was from OriginLab Corporation (Northampton, MA, USA); TMHMM v2.0 was provided by the Center for Biological Sequence Analysis (Technical University of Denmark, Kgs. Lyngby, Denmark, https://services.healthtech.dtu.dk/, accessed on 26 March 2026).

**Table 3:** The advantages and disadvantages of different technologies in PR verification.

Technology	Advantages	Disadvantages
XOE-TEVC	*Xenopus* oocytes are large, facilitating microelectrode insertion and stable recording of electrophysiological signals for easy experimental operation. The expression system has high stability and enables efficient expression of exogenous PR proteins, ensuring sufficient receptor levels for reliable electrophysiological detection. As a classic and mature in vitro validation technique, it has good result reproducibility and is suitable for functional identification of PRs in most insect species. It directly captures electrophysiological responses of PRs after sex pheromone binding, intuitively reflecting the ligand recognition specificity and response intensity of receptors.	Endogenous muscarinic receptors in oocytes may cause non-specific activation, requiring pharmacological blockers and control experiments to eliminate interference, thus increasing experimental procedures. The experimental process relies on precise microinjection techniques, and the cost of professional electrophysiological recording equipment is relatively high. As an in vitro heterologous expression system, it lacks the natural physiological environment of insect bodies and cannot fully simulate the actual functional state of PRs in insect olfactory sensory neurons. Experimental operation requires certain technical proficiency; steps such as microelectrode insertion and signal recording are susceptible to operational errors, which may lead to deviations in results.
*Drosophila* ab3	Lack of endogenous receptors enables low-interference and specific detection of interactions between PRs and sex pheromone ligands. The near-insect physiological environment can accurately simulate the folding state and functional activity of PRs. Relies on mature SSR technology with high detection sensitivity and stability for accurate characterization of PR ligand response characteristics.	The cross-order interaction efficiency between PRs from non-dipteran insects and *Drosophila* Orco may be limited in some species, and functional complexes may fail to form. The detection sensitivity of SSR is relatively low, making it prone to missing low-affinity PR–ligand interactions. Lipophilic sex pheromones are difficult to precisely control in terms of concentration and action time in the experimental system, affecting the quantitative accuracy of results.
Cell Line Heterologous Expression System	Allows efficient exogenous PR expression via optimized culture conditions, ensuring adequate functional receptor production for subsequent detection. Enables real-time and dynamic monitoring of PR activation processes when combined with calcium imaging and other technologies. Provides a post-translational modification environment close to the native state, facilitating correct PR folding and maintenance of functional activity. Offers diverse cell line options (insect/mammalian) for flexible selection based on experimental purposes and PR intrinsic traits.	1. Mammalian cell lines lack the natural physiological background of insects, which may lead to differences in PR functional performance. 2. The experimental operation involves multiple steps such as cell transfection and culture, with strict requirements for experimental conditions and operation proficiency. 3. Part of PRs may have low expression efficiency or poor solubility in heterologous cells, affecting subsequent functional verification. 4. The detection process relies on specialized instruments and fluorescent indicators, resulting in relatively high overall experimental costs.
RNA Interference	Enables highly specific silencing of target *PR* genes, minimizing off-target interference with non-target genes and ensuring accurate functional analysis. Features flexible dsRNA delivery routes (microinjection, oral feeding, topical application) to adapt to experimental needs of different insect species. Realizes in vivo verification of PR functions by monitoring behavioral changes after gene silencing, reflecting the actual regulatory role of PRs in insect mating. Possesses good environmental compatibility, as dsRNA degrades easily in nature without causing persistent ecological pollution, aligning with green pest control concepts.	1. dsRNA is susceptible to degradation by endogenous and environmental nucleases, leading to unstable silencing efficiency and short effective duration of gene suppression. 2. Exists significant interspecific differences in dsRNA absorption efficiency; some insect species have intestinal barriers that hinder dsRNA from reaching target tissues, limiting silencing effects. 3. Often requires repeated dsRNA delivery to maintain effective *PR* gene silencing. 4. Only achieves transient gene silencing rather than stable genetic modification, making it unsuitable for long-term functional research of *PR* genes. 5. Silencing efficiency is easily affected by insect developmental stages and tissue types, leading to inconsistent experimental results across different test conditions.
CRISPR/Cas	Enables high-efficiency targeted editing of *PR* genes, rapidly constructing PR function-deficient insect strains for in vivo functional validation. Possesses high editing precision; optimized sgRNA design effectively reduces off-target risks, ensuring the accuracy of phenotypic analysis of *PR* gene knockout. The editing effect is stably heritable, facilitating long-term research on *PR* gene functions and evolutionary mechanisms across insect generations. Realizes diverse genetic modifications (knockout, knock-in, site-directed mutagenesis), allowing in-depth exploration of key amino acid sites and structural domains of PRs.	Off-target effects cannot be completely eliminated, and unintended genetic mutations may cause abnormal phenotypes, interfering with the analysis of *PR* gene functions. The technical system is immature for many non-model insect species; embryo microinjection and other operations are difficult and have low success rates. The experimental cycle is long, and the operation is complex, involving sgRNA design, microinjection, screening of positive strains and other multiple steps, with high technical requirements for researchers. High experimental cost, including the cost of vector construction, microinjection equipment and insect rearing, is not suitable for large-scale screening of *PR* genes. For some *PR* genes with functional redundancy, single-gene knockout may not produce obvious phenotypic changes, leading to the failure of functional verification.

## Data Availability

No new data were created or analyzed in this study. Data sharing is not applicable to this article.
